# Malunion der distalen Radiusfraktur: 3-D-Planung und Durchführung von intra- und extraartikulären Korrekturosteotomien

**DOI:** 10.1007/s00064-023-00808-8

**Published:** 2023-05-02

**Authors:** Raffael Labèr, Andreas Schweizer

**Affiliations:** Handchirurgie, Universitätsklinik Balgrist, Forchstr. 340, 8008 Zürich, Schweiz

**Keywords:** Intraartikulär, Osteotomie, 3‑D-Planung, 3‑D-geführte Operation, Schablonen, Intraarticular, Osteotomy, Three-dimensional planning, 3D-guided surgery, Guides

## Abstract

**Operationsziel:**

Wiederherstellung der ursprünglichen Anatomie mit Reduktion des aktuellen Beschwerdebildes sowie Risikominderung einer posttraumatischen Arthrose.

**Indikationen:**

Symptomatische intra- oder extraartikuläre Malunion aufgrund von Bewegungseinschränkung und/oder schmerzhafter Funktion, intraartikuläre Stufe von > 1 mm, Instabilität distales Radioulnargelenk.

**Kontraindikationen:**

Minimale Fehlstellung. Bereits bestehende Arthrose Knirk und Jupiter II oder höher. Einfachere operative Alternative z. B. Ulnaverkürzungsosteotomie. Raucher oder erhöhtes Alter stellen keine Kontraindikation dar.

**Operationstechnik:**

Präoperative Befunderhebung und Durchführung eines beidseitigen Computertomogramms. 3‑D-Fehlstellungsanalyse und Berechnung einer Korrektur. Planung der Korrekturosteotomie am 3‑D-Modell und Erstellung von patientenspezifischen Bohr- und Sägeschablonen. Durchführen der 3‑D-gestützten Osteotomie.

**Weiterbehandlung:**

Frühfunktionelle unbelastete Mobilisation aus der Schiene für 8 Wochen bis zur Konsolidationskontrolle mit Computertomographie.

**Ergebnisse:**

Reduktion der Stufe auf < 1 mm (*p* = < 0,05) kann bei intraartikulären Korrekturen erreicht werden. Bei extraartikulären Korrekturen können im Mittel der resultierende Rotationsfehler von 2,0° (± 2,2°) und ein Translationsfehler von 0,6 mm (± 0,2 mm) erreicht werden. Einzelschnittosteotomien im Schaftbereich können auf wenige Grad genau erfolgen für Rotation (z. B. Pronation/Supination 4,9°) und für die Translation (z. B. proximal/distal 0,8 mm). Eine residuelle Abweichung des kombinierten 3‑dimenisionalen Winkels von 5,8° (SD 3,6°) wird festgehalten. Weiter zeigt die 3‑D-gestützte Operation eine signifikante Reduktion der Operationszeit im Vergleich zur konventionellen Technik (140 ± 37 vs. 108 ± 26 min [*p* < 0,05]). Somit kann die Progression einer Arthrose im mittelfristigen Verlauf reduziert werden, und eine gebesserte Beweglichkeit und Faustschlusskraft werden erreicht. Die klinischen Outcome-Parameter des Patient-Rated Wrist Evaluation (PRWE) Score und des Disablities of the Arm, Shoulder and Hand (DASH) Score sind in etwa vergleichbar.

## Abstract (en)

**Objective:**

Restoration of the original anatomy with reduction of both current symptoms and risk of posttraumatic osteoarthritis.

**Indications:**

Symptomatic intra- or extra-articular malunion due to limitation of movement and/or painful function, intra-articular step of > 1 mm, instability of the distal radioulnar joint.

**Contraindications:**

Minimal deformity. Pre-existing osteoarthritis Knirk and Jupiter II or higher. Simpler surgical alternative, e.g., ulna shortening osteotomy. Smoking or advanced age are not contraindications.

**Surgical technique:**

Preoperative assessment and performance of a bilateral computed tomography (CT). Three-dimensional (3D) malposition analysis and calculation of the correction. Planning of the corrective osteotomy on the 3D model and creation of patient-specific drilling and sawing guides. Performing the 3D-guided osteotomy.

**Postoperative management:**

Early functional unloaded mobilization with the splint for 8 weeks until consolidation control with CT.

**Results:**

Significant reduction of the step to < 1 mm (*p* ≤ 0.05) can be achieved with intra-articular corrections. In extra-articular corrective osteotomies, a mean residual rotational malalignment error of 2.0° (± 2.2°) and a translational malalignment error of 0.6 mm (± 0.2 mm) is achieved. Single-cut osteotomies in the shaft region can be performed to within a few degrees for rotation (e.g., pronation/supination 4.9°) and for translation (e.g., proximal/distal, 0.8 mm). After surgery, a mean residual 3D angle of 5.8° (SD 3.6°) was measured. Furthermore, surgical time for 3D-assisted surgery is significantly reduced compared to the conventional technique (140 ± 37 vs 108 ± 26 min; *p* < 0.05). Thus, the progression of osteoarthritis can be reduced in the medium term and improved mobility and grip strength are achieved. The clinical outcome parameters based on patient-rated wrist evaluation (PRWE) and the disabilities of the arm, shoulder and hand (DASH) scores are roughly comparable.

## Vorbemerkungen

Trotz fortschrittlicher bildgebender Verfahren und optimierter Frakturversorgung sind fehlverheilte Frakturen (Malunionen) des distalen Radius häufig [2, 3]. Die Malunion kann naturgemäß intra- wie extraartikulär auftreten und entsprechend die Beweglichkeit einschränken und Instabilität sowie Schmerzen zur Folge haben [4]. Über Jahrzehnte waren Korrekturosteotomien im Allgemeinen nur an 2 in etwa 90° zueinanderstehenden konventionellen Röntgenbildern planbar. So musste ein fehlgestellter Körper (Frakturelement) in einem aus 3 Achsen bestehenden Koordinatensystem anhand eines 2‑Achsen-Modells korrigiert werden, was naturgemäß die 3. Ebene vernachlässigte, sprich bei einer distalen Radiusmalunion kann in der Achse von Flexion/Extension sowie in der Achse Radial/Ulnaduktion korrigiert werden, jedoch nicht in der Längsachse im Sinne von Pro‑/Supination. Mit der Computertomographie (CT) und entsprechender Software kann nun die Fehlstellung in einem korrekten 3‑Achsen-Koordinatensystem abgebildet, analysiert und korrigiert werden. Dies erfolgt in einem ersten Schritt über die Segmentierung der CT-Bilder. Hier werden benachbarte homogene Bildpunkte (Voxel), welche dem Knochen entsprechen, zusammengefügt. So lässt sich ein über die CT-Schnitte definiertes 3‑dimensionales Objekt erstellen. Der so erhaltene Knochen kann über das identisch erstellte kontralaterale, gespiegelte Knochenmodell überlagert werden. Dies führt zu einer direkten visuell sichtbaren Differenz beider Knochen zueinander. Die Differenzen, respektive die somit vorliegende Fehlstellung, können im Koordinatensystem noch mittels exakter Zahlen quantifiziert werden. Somit lassen sich einfache wie auch komplexere Fehlstellungen nun millimeter- sowie gradgenau korrigieren [8]. Verschiedene Techniken, basierend auf 3‑D-Analysen und Planungen, wurden in den letzten Jahren publiziert [5, 6, 10]. Hier möchten wir mögliche 3‑D-Analysen und Planungen sowie bis anhin nicht veröffentlichte Repositionsschablonen (Repositionsguides) präsentieren.

## Operationsprinzip und -ziel

Das Ziel jeder Korrekturosteotomie ist die Wiederherstellung einer möglichst genauen knöchernen Anatomie mit Optimierung der Klinik im Hinblick auf Funktion, Stabilität, Schmerz und Langzeitverlauf.

## Vorteile

- Komplexe Korrektur extra- sowie intraartikulär
- Exakte Korrektur auf Grad (°) und Millimeter (mm)
- Kürzere Operationszeit
- Technisch einfacher durch exakte Vorgabe der Schnittebenen und Reposition mittels Schablonen

## Nachteile

- Zeitaufwendige Planung (2–4 h je nach Komplexität)
- Kosten
- Leicht größere Zugänge

## Indikationen

- Funktional einschränkende Fehlstellung (Angulation, Rotation, Translation)
- Instabilität distales Radioulnargelenk
- Gelenkstufe: ≥ 1 mm
- Schmerzen
- Beckenkammspongiosa bei aufklappenden/verlängernden Korrekturen, Allograft wird aufgrund der Biologie und Kosten nicht verwendet

## Kontraindikationen

- Minimale Fehlstellung, unter dem möglichen Korrekturausmaß
- Arthrose (Knirk und Jupiter Grad II oder höher) ist individuell zu beurteilen, z. B. intraartikuläre Stufe mit direkt lokaler Schleifspur stellt keine Kontraindikation dar
- Alternative einfachere Operation, z. B. Ulnaverkürzungsosteotomie im Falle einer in erster Linie verkürzten Radiusmalunion mit DRUG-Instabilität ohne relevante Begleitfehlstellungen am Radius

## Patientenaufklärung

- Allgemeine Operationsrisiken: z. B. Infektionen, Wundheilungsstörungen, Nachblutungen
- Pseudarthrose
- Lange Heilungsdauer: > 8 Wochen
- Gegebenenfalls notwendige autologe Spongiosaentnahme (Beckenkamm)
- Restbeschwerden
- Temporäre/persistierende Bewegungseinschränkung
- Fraktur Beckenkamm
- Drainageneinlage
- Folgeeingriffe (Metallentfernung, Tenolysen)
- CRPS
- Hospitalisation von 2 Nächten
- Arbeitsunfähigkeit ca. 4 bis 6 Wochen (administrativ), ca. 12 Wochen (körperlich)
- Ruhigstellung in Gipsschiene mit erlaubter unbelasteter Mobilisation aus der Schiene für 8 Wochen bis zur Konsolidationskontrolle mit Computertomographie

## Operationsvorbereitungen

- Klinische Untersuchung und Dokumentation (ROM, DRUG-Stabilität, Faustschlusskraft)
- Konditionierung der Weichteile
- Computertomographien beider Unterarme
- Segmentierung der CT-Bilder zur Erstellung eines 3‑D-Knochenmodells beider Unterarme
- Spiegeln des gesunden kontralateralen 3‑D-Knochenmodells, sodass eine Überlagerung möglich wird
- Fehlstellungsanalyse mittels Software (z. B. CARD©, Balgrist) in dem 3‑D-Knochenmodelle überlagert werden und die Differenzen quantifiziert werden können
- Planung der Korrektur am 3‑D-Modell
- Planung der Bohr- und Sägelehren mittels Negativabdruck am Knochen mit spezifischen Referenzpunkten
- 3‑D-Druck der Knochenmodelle sowie Bohr- und Sägelehren mittels z. B. EOS Formiga P100 printer (Electro Optical Systems GmbH©, Krailling, Germany) mit PA 2200 (polyamid-12) Pulver (Electro Optical Systems GmbH©, Krailling, Germany) über ein selektives Laser-Sintering(SLS)-Verfahren
- Initiales Testen der Schablonen am Modellknochen unsteril, sofern guter und korrekter Sitz der Schablonen, erfolgt die Freigabe zur Sterilisation
- Implantat zur Fixation bestellen (z. B. Intercus Correctus Platte©, Intercus Schweiz, 5000 Aarau, Schweiz)
- Rasur im Operationsgebiet (Vorderarm/Beckenkamm)
- Antibiotikaprophylaxe mittels Einzeldosis präoperativ

## Instrumentarium

- Knochenmodell und Guides (Sägelehre/Schnittblöcke/Schablonen)
- Bohrhülsen aus dem 3‑D-Drucker oder aus Metall
- Kanülierte Meißel
- Gewinde- und Standard-Kirschner-Drähte

## Anästhesie und Lagerung

- Plexusanästhesie und/oder Intubationsnarkose (cave: Beckenkammentnahme)
- Rückenlage mit Handtisch
- Oberarmblutsperre
- Waschen und Abdecken auch an der Entnahmestelle am Beckenkamm
- Perioperative Antibiose (z. B. Cefuroxim)

## Operationstechnik

(Abb. 1, 2, 3, 4, 5, 6, 7, 8, 9 und 10)

**Figure 1:**
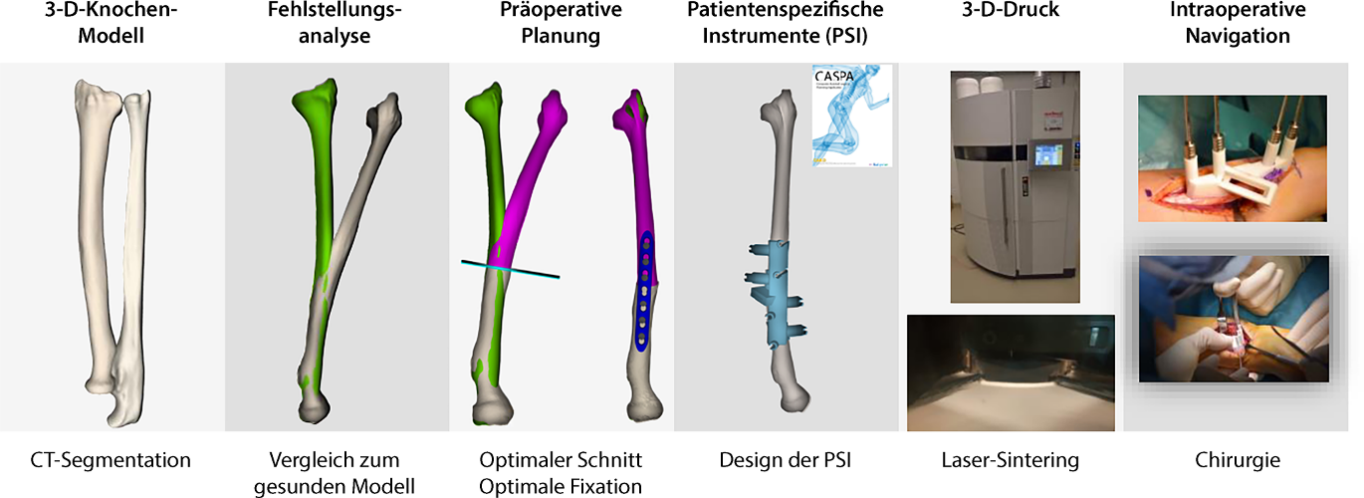

**Figure 2:**
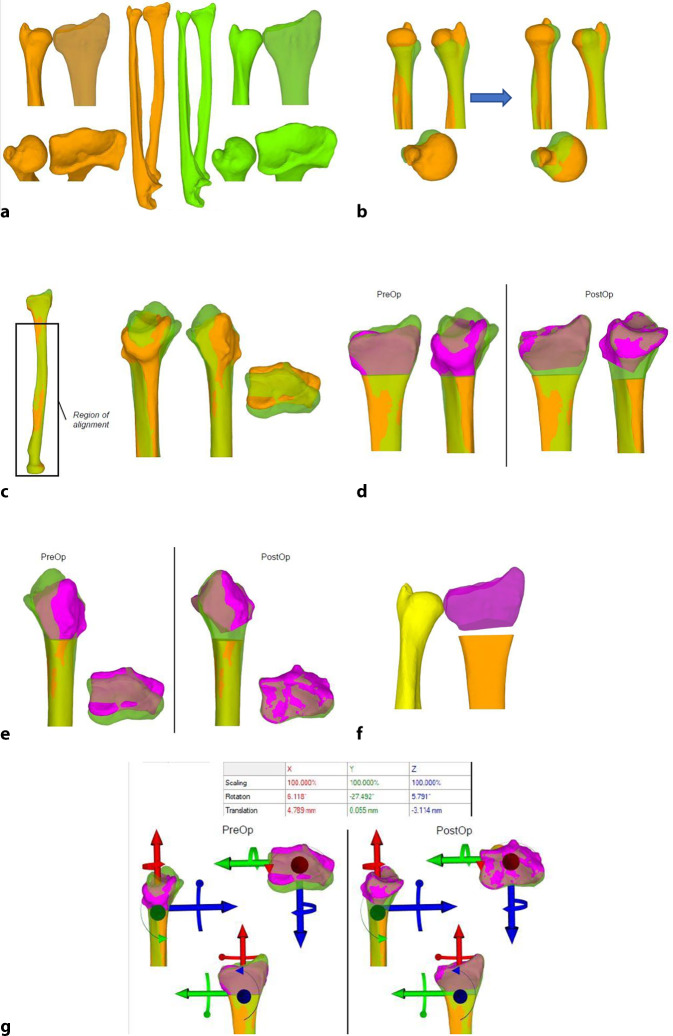

**Figure 3:**
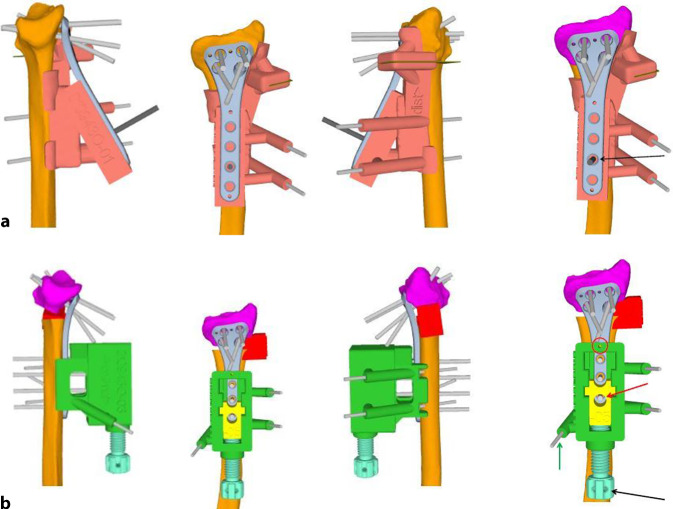

**Figure 4:**
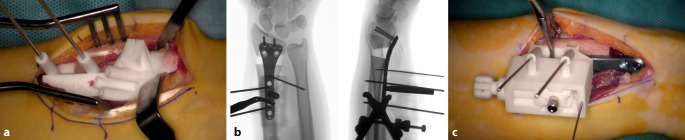

**Figure 5:**
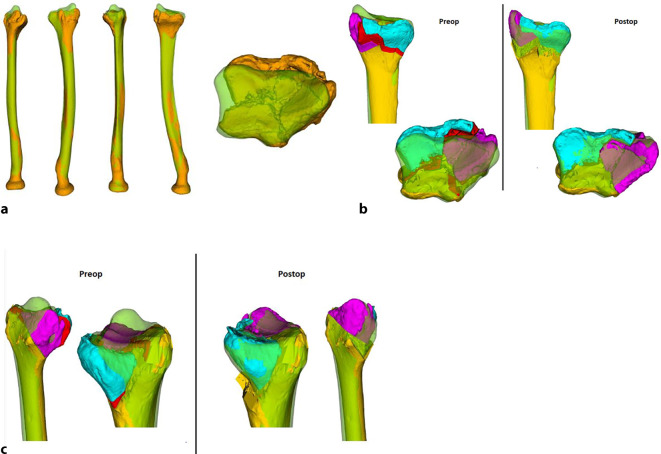

**Figure 6:**
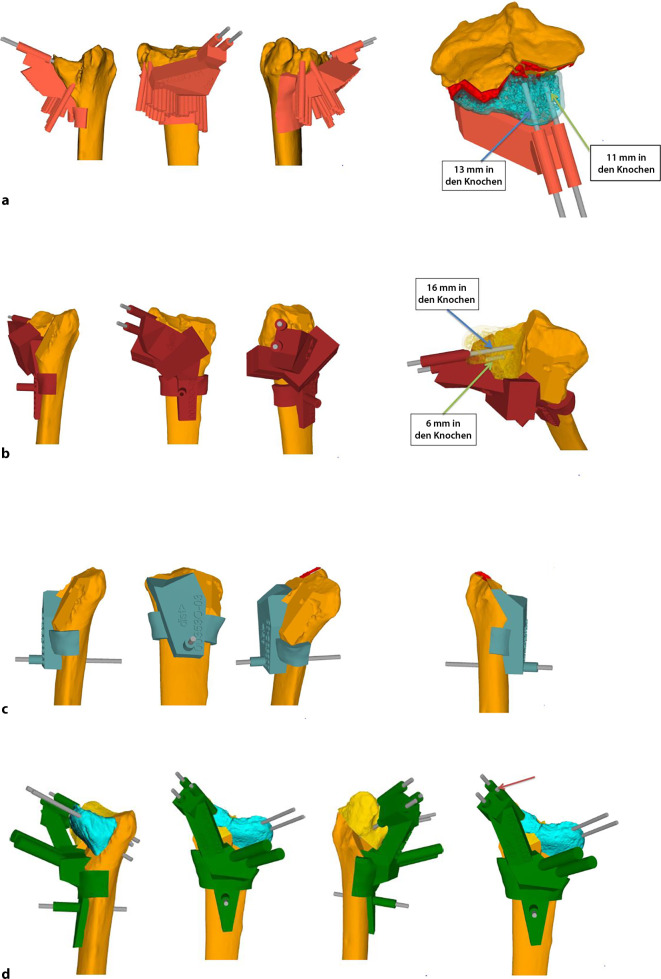

**Figure 7:**
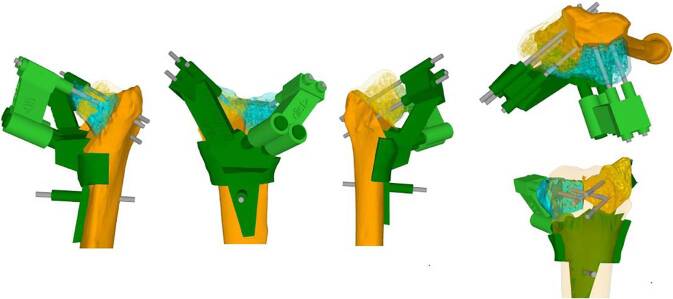

**Figure 8:**
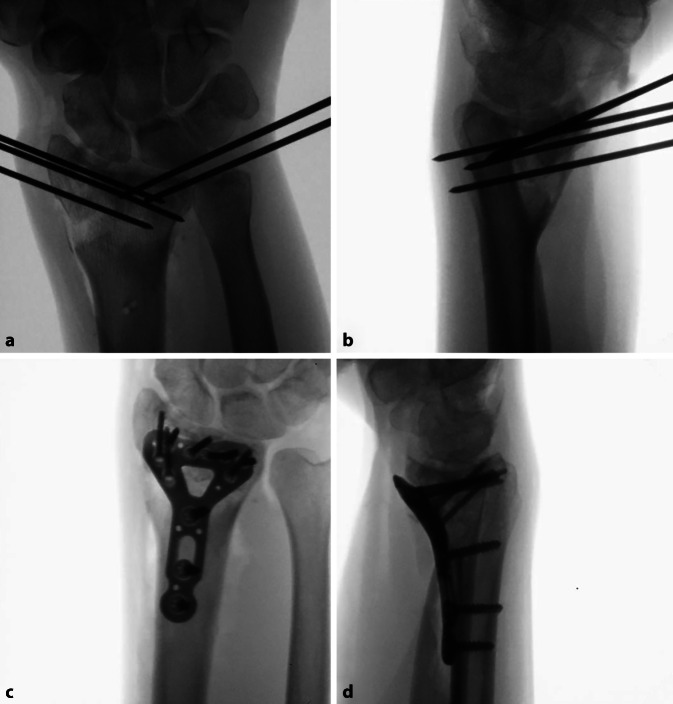

**Figure 9:**
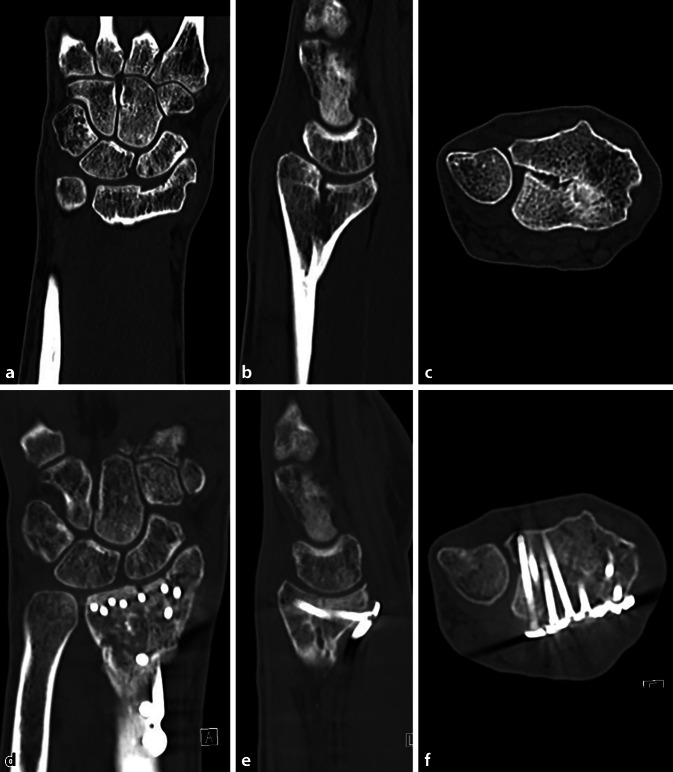

**Figure 10:**
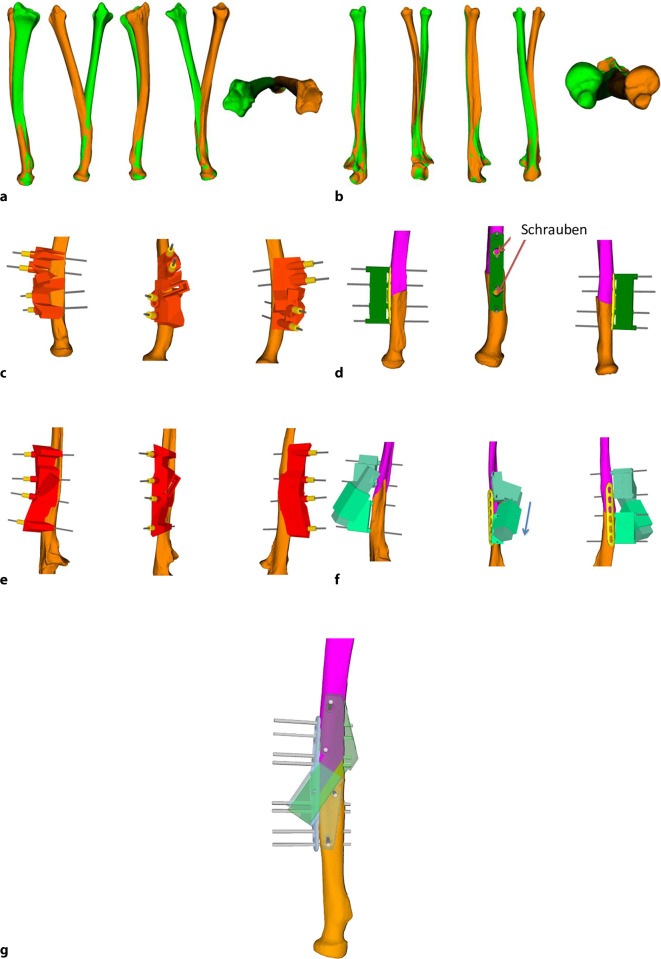

## Postoperative Behandlung

- Wundverband mittels Steristrips und Kompressen
- Steristrips und Comfeel-Verband am Beckenkamm
- Palmare Gipsschiene direkt im OP angepasst
- Drainagenzug am 1. postoperativen Tag
- Sofortige funktionelle unbelastete Mobilisation aus der Gipsschiene, angeleitet durch die Ergotherapie
- Klinische Kontrolle und Fadenmaterialentfernung 12 bis 14 Tage postoperativ
- Anpassen einer thermoplastischen Unterarmschiene für weitere 6 Wochen
- Klinische und computertomographische Kontrolle 8 Wochen postoperativ
- Fortgeschrittene Konsolidation nachweislich → Schienenentwöhnung und Beginn einer Kräftigung
- Erneute computertomographische oder konventionell radiologische Kontrolle 3 Monate postoperativ
- Konsolidation erreicht → Freigabe zur Maximalbelastung
- Klinisch-radiologische Kontrolle mit konventionellem Röntgen nach 6 und 12 Monaten postoperativ
- Planung Entfernung des Osteosynthesematerials, sofern angezeigt, frühestens ab 12 Monate postoperativ distaler Radius und 18 Monate Schaftbereich
- Durchschnittliche Heilungsdauer zwischen 8 und 12 Wochen
- Arbeitsunfähigkeit für administrative Berufe ca. 2 bis 4 Wochen, für manuelle/belastende Berufe mindestens 12 Wochen

## Fehler, Gefahren, Komplikationen

- Schlecht sitzende Schnittblöcke: korrekte Positionierung erschwert → Konturierung des Knochens anhand Knochenmodell (cave: Remodelling)
- Pseudarthrose: Revisionseingriff nach entsprechendem Zeitraum
- Repositionsverlust: sofern symptomatisch oder relevante Fehlstellung → sofern vertretbar ausheilen lassen und Re-Korrekturosteotomie im Verlauf oder sofortige Korrektur unter Verwendung adäquater Fixationstechnik und Implantate
- Infekt: Standardvorgehen bei operationsassoziiertem Infekt

## Ergebnisse

In unserer Institution werden bereits seit über einem Jahrzehnt mittels 3‑D-Analysen Knochenmodelle erstellt und Korrekturen berechnet und geplant. Eine entsprechende Entwicklung in den notwendigen Softwares sowie Computertomographieprotokollen hat die Ergebnisqualität weiter verbessert.

In einer hauseigenen Studie über die intraartikulären Korrekturosteotomien, welche von 2008 bis 2016 durchgeführt wurde, konnten folgende Ergebnisse erzielt werden: 37 Patienten wurden inkludiert, welche eine minimale Gelenkstufe von > 1 mm aufwiesen und eine symptomatische, eingeschränkte Beweglichkeit, ein instabiles distales Radioulnargelenk oder Handgelenksschmerzen hatten. Nach erfolgter 3‑D-Analyse und 3‑D-gestützter Korrekturosteotomie zeigte sich eine vollständige Konsolidation bei allen Patienten. Keine schwerwiegenden Komplikationen wie Infektionen oder Knochennekrosen wurden beobachtet. Die präoperative Gelenkstufe war im Schnitt 2,5 mm, diese konnte auf eine Stufe von 0,8 mm reduziert werden (*p* < 0,05). So hatten nach Operation 30 Patienten eine Stufe < 1 mm; bei 7 Patienten fand man eine Stufe von 1,1–1,4 mm. Nach 1 Jahr hatten 22 Patienten keine Schmerzen mehr, 9 hatten wenige Schmerzen bei belastender Arbeit, und 5 hatten wenig Schmerzen sowie keine Verbesserung zum präoperativen Befund, jedoch zeigten sich eine gebesserte Beweglichkeit sowie Faustschlusskraft. Bei einem Patienten musste aufgrund von persistierenden Beschwerden und residueller Gelenkstufe von 0,6 mm eine radioskapholunäre Fusion erfolgen [8]. Singh et al. kontrollierten 15 Patienten in einem mittleren Zeitraum von 6 Jahren (4,1 bis 10,4 Jahren) nach und hielten fest, dass 8 Patienten keine Progression der Arthrose postoperativ aufwiesen; 6 Patienten entwickelten eine um 1 Grad höhere Arthrose und 2 Patienten um 2 Grad nach Knirk und Jupiter. Ein zweiter unabhängiger Untersucher klassifizierte 11 Patienten als progressionslos, 2 Patienten mit einer Progression um 1 sowie 2 Patienten um je 2 Grad. Auch dort konnte eine gebesserte Faustschlusskraft von + 14,8 kg (± 12,6 kg) dokumentiert werden. Die klinischen Outcome-Parameter waren wie folgt: Der Patient-Rated Wrist Evaluation (PRWE) Score lag bei 11,8 (± 12,0), der mittlere Disabilities of the Arm, Shoulder and Hand (DASH) Score bei 11,1 (± 11,4) und der mittlere Schmerzwert auf der analogen Schmerzskala bei 0,8 (± 1,0) [11].

Weiter konnte unsere Gruppe bei extraartikulären aufklappenden Osteotomien mittels eines dezidierten Guides im Durchschnitt einen deutlich gebesserten restlichen Rotationsfehlwinkel von 2,0° (± 2,2°) und einen Translationsfehler von 0,6 mm (± 0,2 mm), verglichen mit 4,2° (± 15,0°) und 1,0 mm (± 0,4 mm), in der Kontrollgruppe beobachten. Die verwendete Platte wurde nicht signifikant genauer positioniert, aber es wurden signifikant weniger Schrauben (15,6 %) im distalen Fragment falsch ausgerichtet als in der Kontrollgruppe (51,9 %) [7].

In einer weiteren Studie konnte die Genauigkeit auch für Single-Cut-Osteotomien im Schaftbereich bestätigt werden. So zeigten Roner et al. 2017, dass die Korrektur einen mittleren residuellen Fehler in Bezug auf die Rotation (Pronation on/Supination 4,9°, Flexion/Extension 1,7°; ulnar/radial Angulation 2,0°) und für die Translation (proximal/distal, 0,8 mm; radial/ulnar, 0,8 mm; dorsal/palmar, 0,8 mm) aufweist. Eine mittlere residuelle Abweichung des kombinierten 3‑dimenisionalen Winkels von 5,8° (SD 3,6°) wurde festgestellt. Alle 6 Patienten, die aufgrund eingeschränkter Beweglichkeit in der Umwendbewegung operiert wurden, erreichten eine Symptomreduktion und gebesserte Beweglichkeit von 20 auf 80°. Bei den Patienten mit instabilem oder schmerzhaftem DRUG waren 3 Patienten schmerzfrei, und einer hatte noch bei sportlicher Aktivität Beschwerden [9]. In allen Studien konnte eine niedrige Pseudarthrosenrate festgehalten werden [7–9, 11, 12].

Weiter konnte in einer Vergleichsstudie zwischen der konventionellen (*n* = 31) und computerassistierten (*n* = 25) Methode einer Korrekturosteotomie eine deutlich kürzere Operationszeit 140 ± 37 vs. 108 ± 26 min (*p* < 0,05) nachgewiesen werden. Weiter konnte nach 12 Wochen in der computerassistierten Kohorte eine höhere Anzahl an konsolidierten Osteotomien festgehalten werden (60,9 % vs. 32,3 %, *p* < 0,05). Zwei Patienten aus der computerassistierten Kohorte benötigten einen Revisionseingriff aufgrund einer Non-Union. Die klinischen Outcome-Parameter waren in den gematchten Gruppen identisch [1].
